# Severe anaemia secondary to a perforated gastric ulcer in a male alpaca

**DOI:** 10.1186/s13620-023-00251-y

**Published:** 2023-09-09

**Authors:** Matthias Gerhard Wagener, Teresa Maria Punsmann, Sven Kleinschmidt, Ralf Surholt, Saskia Neubert, Hannah Marahrens, Thekla Großmann, Martin Ganter

**Affiliations:** 1 Clinic for Swine and Small Ruminants, Forensic Medicine and Ambulatory Service, University of Veterinary Medicine Hannover, Foundation, 30173 Hannover, Germany; 2 Lower Saxony State Office for Consumer Protection and Food Safety, Food and Veterinary Institute Braunschweig/Hannover, 30173 Hannover, Germany; 3 Tierarztpraxis Dr. Ralf Surholt, 27639 Midlum, Wurster Nordseeküste Germany

**Keywords:** South American camelids, Gastric ulcer, Anaemia, Colic, Peritonitis

## Abstract

**Background:**

Anaemia is a common condition in alpacas and attributable to a variety of causes. Severe anaemia with a packed cell volume (PCV) less than 10% is frequently diagnosed, usually due to blood loss resulting from haemonchosis. Many South American camelids (SACs) also suffer from gastric ulcers, which are often associated with anaemia in other species. However, in alpacas and llamas, gastric ulcers usually do not lead to anaemia due to blood loss according to the current literature. There are no detailed clinical and laboratory data on this condition in the scientific literature so far.

**Case presentation:**

We report on the case of a nine-year-old male alpaca that was presented to the clinic with suspected forestomach acidosis. The animal showed clinical signs of colic, hypothermia, tachypnea, tachycardia, pale mucous membranes, and died shortly after admission to the clinic. Laboratory diagnosis revealed a markedly decreased haematocrit (0.13 l/l), leucopaenia with band neutrophils, azotaemia, hypocalcaemia, hyperphosphataemia and vitamin D deficiency. Post-mortem examination revealed multiple ulcers in the first and third compartment with perforation of one ulcer in the first compartment, resulting in intraluminal blood loss and purulent peritonitis.

**Conclusions:**

To the authors’ knowledge, this is the first detailed description of clinical and laboratory data of severe anaemia due to a perforated gastric ulcer in a SAC. Although the current literature suggests that severe blood loss due to gastric ulcers does not occur in SACs, this condition should be considered as a possible differential diagnosis in anaemic animals. Clinical indicators can be colic and pale mucous membranes.

## Background

Anaemia is a common condition in alpacas and llamas [1]. About half of all South American camelids (SACs) presented to the Clinic for Swine and Small Ruminants, University of Veterinary Medicine Hannover, Foundation, Hannover, Germany reveal a packed cell volume (PCV) below the reference range [2, 3]. Various data on haematological reference ranges in alpacas can be found in the literature [3–5]. Although there is no general classification of the severity of anaemia in SACs, the suggestions from Wittek and Franz can be consulted: PCV: 0.25–0.20 l/l: mild anaemia; PCV: 0.20–0.15 l/l: moderate anaemia; PCV: 0.15–0.10 l/l: severe anaemia; PCV < 0.10 l/l: fatal anaemia [6]. In the case of fatal anaemia, blood transfusion is usually necessary as an immediate life-saving intervention [6].

There are different causes of anaemia in SACs. Severe or fatal anaemia is often associated with endoparasitosis caused by *Haemonchus contortus* [7–9]. Anaemia in those cases occurs due to attachment of female adult worms to the mucosa of the third compartment, as one adult worm can result in blood losses of approximately 50 µl of blood daily [10, 11]. In an infestation of 1,000 worms, the daily blood loss would be approximately 50 ml.

Another pathogen associated with anaemia in alpacas and llamas is *Candidatus Mycoplasma haemolamae* [12]. The mycoplasms infect erythrocytes and are regularly found in the blood of SACs, but can also cause asymptomatic infections [13–16].

In addition to the aforementioned causes of regenerative anaemia, non-regenerative anaemia in SACs may be associated with iron, cobalt or copper deficiency [17–19].

Gastric ulcers, which occur regularly in alpacas and llamas [20, 21], can also result in anaemia, as the mucosa of the third compartment is well vascularised in SACs [22]. The structure of the stomach of camelids differs anatomically from that of ruminants, with a division into three compartments (C1, C2, C3) [23]. While C1 and C2 correspond to the forestomach, the distal part of the C3 can be compared to the abomasum of ruminants. Unlike the abomasum, the C3 has an elongated and tubular form. A detailed description of the anatomy of the compartments in alpacas can be found in Vater et al. [23].

In contrast to cattle, in which abomasal ulcers often result in anaemia [24, 25], ulcers of the third compartment in SACs are rarely associated with severe or fatal anaemia [19, 21]. Although some authors mention anaemia as a clinical finding in gastric ulcers in SACs [12], there are currently no specific descriptions in the scientific literature of individual cases of SAC with severe anaemia caused by gastric ulcers.

This case presentation describes laboratory and pathological findings in a male alpaca with multiple gastric ulcers and severe anaemia secondary to a perforated ulcer of the first compartment discussed within the context of existing literature.

## Case presentation

### Anamnesis and pretreatment

In January 2021, a nine-year-old, 66-kg male alpaca from a herd of approximately 30 alpacas was presented to the Clinic for Swine and Small Ruminants due to suspected forestomach acidosis. The owners presumed that the animal may have ingested too many pears on the previous day. They noticed pale mucous membranes, hypothermia and colic symptoms. On the same day, pretreatments were already performed by the veterinary practice on site. Firstly a spasmolytic and NSAID (75.8 mg/kg body weight (bw) metamizole sodium and 0.6 mg/kg butylscopolaminium bromide: Buscopan compositum®, Boehringer-Ingelheim Vetmedica GmbH, Ingelheim, Germany), 0.21 mg/kg bw dexamethasone (Rapidexon Albrecht 2 mg/ml, Dechra Veterinary Products Deutschland GmbH, Aulendorf, Germany), as well as infusions with 500 ml of physiological NaCl solution and 125 ml of sodium bicarbonate (8.4%) were administered. A blood sample revealed severe anaemia (PCV: 0.13 l/l) and hyperglycaemia, therefore a blood transfusion with 500 ml of whole blood from a healthy donor from the same herd was performed, furthermore insulin was administered systemically. An oral supplement containing B-vitamins was supplemented orally. The animal owners had obtained an exemption permit from their responsible veterinary office for this animal, which allowed the use of medications for non-food-producing animals.

After pretreatment, the animal’s condition improved, but only for a short time. On the following day, the animal’s condition deteriorated again so that the alpaca was presented to the clinic.

### Clinical examination

On general examination at the clinic, the alpaca was recumbent, swaying his head and partially rolling on his back in the box, but showed alert behaviour. Rectal temperature was 35.7 °C, respiratory rate was 56 breaths per minute, heart rate 140 beats per minute. Auscultation of the lungs and heart did not reveal any abnormal findings; auscultation of the first compartment revealed one contraction in two minutes. Neither defaecation nor urination occurred during the examination, bodily condition was good with a BCS of 3.5 [26]. The cornea of the stallion appeared clear, the colour of the conjunctivae was slightly pale (FAMACHA©-score: 2 [27]), the episcleral vessels were finely marked. Palpation of the lymph nodes (*Lnn. cervicales superficiales* and *Lnn. Subiliaci*) did not reveal any abnormal findings. The larynx of the alpaca was symmetrical, a swallowing reflex was elicited, a cough reflex was not. Palpation of the thorax and limbs revealed no abnormalities, the abdominal wall did not reveal increased tension, but was sensitive to palpation. However, the condition of the alpaca continued to deteriorate and the animal started to breathe through its mouth.

### Treatment

Due to the animal’s poor general condition, a venous catheter was inserted in the jugular vein immediately, through which 1.2 mg/kg bw dexamethasone (Dexamethasone 4 mg/ml, Bela-Pharm GmbH & Co. KG, Vechta, Germany) for treatment of a suspected shock, 53.0 mg/kg bw metamizole sodium, 0.42 mg/kg bw butylscopolaminium bromide (Spasmium comp., Richter Pharma AG, Wels, Austria) for treatment of colic symptoms and 0.91 mg/kg bw doxapram hydrochloride (Doxapram-V, Dechra Veterinary Products Deutschland GmbH, Aulendorf, Germany) for respiratory stimulation were administered.

However, treatment did not result in clinical improvement and the animal died within two hours of arrival at the clinical.

### Laboratory findings

In a pooled faecal sample of the male alpacas of the herd (n = 13 animals at this time), which had been taken five days earlier, no eggs of gastrointestinal nematodes could be detected by flotation. There was also no evidence of coccidial oocysts, eggs of *Trichuris* spp., *Capillaria* spp., *Strongyloides* spp. nor evidence of eggs of small and large liver flukes in the sedimentation procedure. An examination with a modified Baermann technique revealed no larvae of small or large lungworms [28].

The results of a venous blood sample taken shortly after presentation to the clinic and analysed in the clinic’s laboratory are displayed in Tables 1 and 2. Haematology revealed severe anaemia, leucopaenia with band neutrophils and an increased neutrophil-to-lymphocyte ratio. Cabot rings were seen in some erythrocytes, there was no detectable evidence of haemotrophic mycoplasmas in the blood smear. A further PCR for *C. M. haemolamae* was not conducted.

**Table 1:** Haematological parameters

Parameter	Reference a)	Reference b)	Reference c)	Alpaca
PCV [l/l]	0.29–0.37	0.22–0.45	0.30–0.42	0.13
Haemoglobin [g/l]	127–166	102–193	144–188	62
MCHC [g/l]	414–459	420–490	382–557	477
WBC (counted) [G/l]				5.7
WBC (corrected) [G/l]	9.8–15.8	7.1–18.6	6.0–20.9	5.6
Lymphocytes [%]	15.2–31		18–49	21
PMN [%]	44–70.5		40–73	50
Band neutrophils [%]	0–1			22.5
Metamyelocytes [%]				3
Myelocytes [%]				0
Eosinophils [%]	8–27		0–15	1
Basophils [%]	0–2			0
Monocytes [%]	2–5.5		0–4	2.5
Nucleated red blood cells [%]		0–3		2
Lymphocytes [G/l]	1.7–4.5	1.1–5.5	2.1–6.8	1.17
PMN [G/l]	4.5–9.3	3.5–11.7	2.0–13.3	2.79
Band neutrophils [G/l]	0–0.1	0		1.26
Metamyelocytes [G/l]				0.17
Myelocytes [G/l]				0
Eosinophils [G/l]	1–3.6	0.1–4.3	0–1.9	0.06
Basophils [G/l]	0–0.3	0–0.4		0
Monocytes [G/l]	0.1–0.7	0–1.0		0.14
NLR			0.5–2.9	3.45
Anisocytosis				+
Polychromasia				+
Poikilocytosis				+

References: a) Hengrave Burri et al. [3], b) Dawson et al. [5], c) Hajduk [4]

*PCV* Packed cell volume, *RBC* Red blood cells, *MCHC* Mean corpuscular haemoglobin concentration, *WBC* White blood count, *PMN* Polymorphonuclear neutrophil, *NLR* Neutrophil-to-lymphocyte ratio, Anisocytosis, polychromasia and poikilocytosis were determined semiquantitatively from + (does not appears microscopically in every field of view) to +  +  +  + (more than 50% of erythrocytes are affected)

**Table 2:** Clinical chemistry parameters

Parameter	Reference a)	Reference b)	Alpaca
Total bilirubin [µmol/l]	0.3–1.1	0–1.7	2.51
Creatinine [µmol/l]	97–167	88–212	422
Urea [mmol/l]	5.2–9.7	3.6–10.7	42.6
Calcium [mmol/l]	2.1–2.5	2.05–2.52	1.78
Phosphate [mmol/l]	1.6–3	1.1–2.52	4.81
CK [U/l]	38–232	29–120	805
ASAT [U/l]	150–263	128–308	295
GLDH [U/l]	3.4–29.8	3–19	14
AP [U/l]	34–190	18–113	60

References: a) Hengrave Burri et al. [3], b) Dawson et al. [29]

Clinical chemistry of plasma samples revealed azotaemia, hypocalcaemia, hyperphosphataemia and an increase in plasma activity for creatine kinase (Table 2). The examination of copper and selenium in serum by means of atomic absorption was in the reference range in accordance with the reference values of Stanitznig et al. [30] (copper: 4.6 µmol/l, reference range 4.28–10.86 µmol/l; selenium: 85.6 µg/l, reference range: 40.2–193.7 µg/l).

### Pathological findings

Necropsy was performed at the Lower Saxony State Office for Food and Consumer Protection and Food Safety (LAVES).

At post mortem, a good nutritional condition was confirmed by the presence of macroscopically visible fat reserves. The mucous membranes of the carcass were pale, the lungs showed congestive hyperaemia. In the abdominal cavity, a small amount of ichorous effusion indicated purulent peritonitis. A mild fatty liver was present and the urinary bladder was well filled with species-specific urine.

**Fig. 1:**
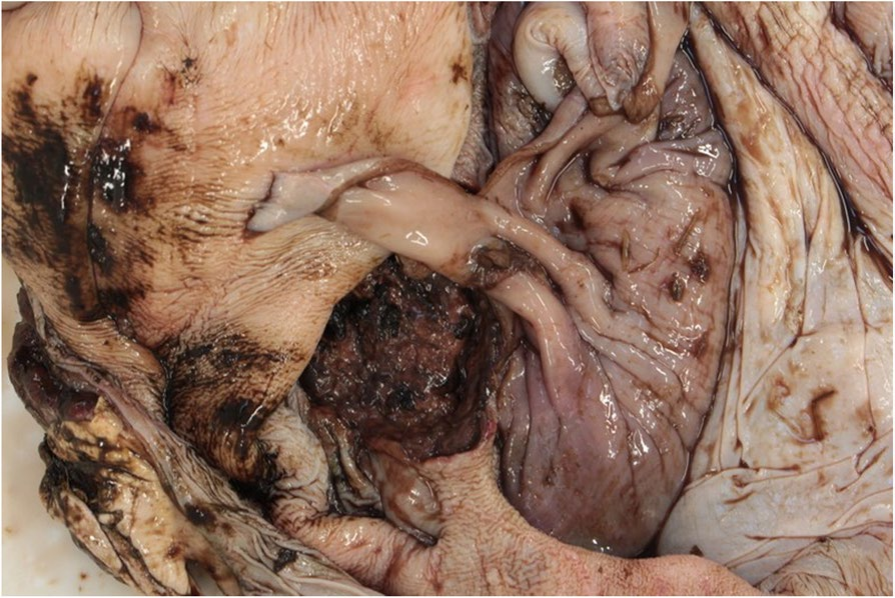
Compartment, focal circular ulcer of approx. 7 cm in diameter with blackish, digested haemorrhages. Surrounding area with multifocal, irregular and different small erosions or ulcerations. © S. Kleinschmidt, LAVES-Hannover

**Fig. 2:**
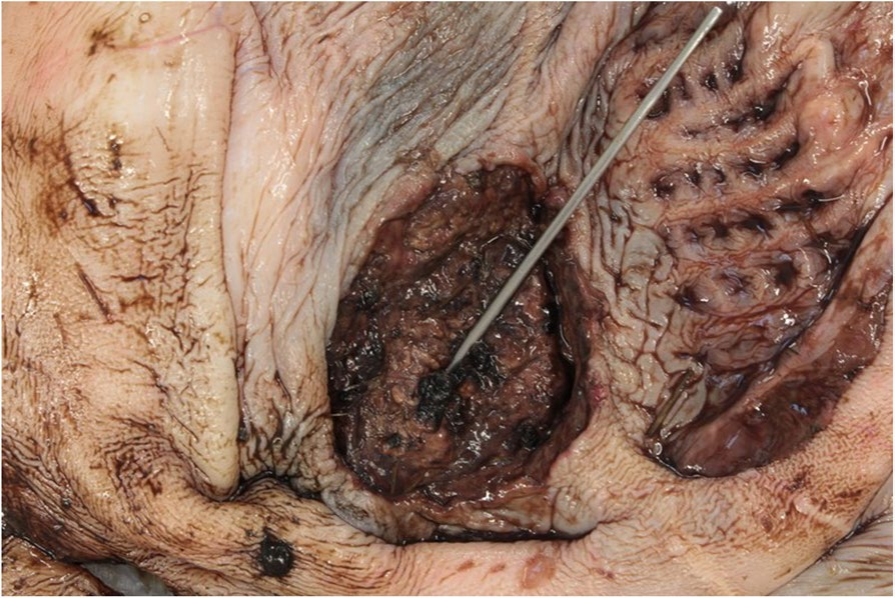
Compartment, close-up of ulcer; probe showing approx. 2 mm in diameter disruption. The raised rim wall indicates the chronicity of the inflammation. © S. Kleinschmidt, LAVES-Hannover

In the first compartment, there was a focal ulcer of 7 cm in diameter, with focal complete disruption of the wall about 2 mm in diameter (Figs. 1 and 2). Furthermore, the third compartment showed multifocal smaller ulcers and erosions.

The contents of the first compartment consisted mainly of animal-specific ground roughage and isolated cereal grains. In the small intestine, there was segmental dark red discoloured ingesta, which indicated digested blood, and the rectal faeces were species-specific in shape.

## Discussion and conclusion

The cause of death in this alpaca was cardiovascular failure in connection with ulcerative, perforated inflammation of the third compartment with consecutive peritonitis. Gastric ulcers are common in alpacas kept in Germany, but perforated gastric ulcers with consecutive peritonitis are rare [21, 31].

Gastric ulcers in alpacas can occur in all compartments, but the third compartment is most frequently affected, as the pH value is lowest there [20, 21, 32]. Stress and the use of non-steroidal anti-inflammatory drugs (NSAIDs) are discussed as triggers [20, 32]. A combination of reduced prostaglandin E synthesis and a simultaneous increase in cortisol, pepsin and gastric acid can lead to damage to the gastric mucosa [20, 32]. In the present case, however, no excessive stressors or NSAID administration were reported in the anamnesis before the veterinary intervention. When the animal was presented for pretreatment, forestomach acidosis was suspected in addition to anemia. The administration of metamizole and dexamethasone to treat the clinical signs of colic at that time were unlikely to be the cause of the perforation of the ulcer, as the alpaca was already presented with anemia and colic symptoms.

Gastric ulcers have also been reported to be associated with reflux of duodenal fluid [33] and in connection with *Eimeria macusaniensis* infections [34]. Gastric ulcers in SAC can further promote other diseases, as they could be a portal of entry for mycoses, for example [35, 36]. There was no evidence for any of these conditions in the animal presented here.

In a recently published retrospective study on gastric ulcers in alpacas that died or were euthanised at the Clinic for Swine and Small Ruminants, Neubert et al. found gastric ulcers in 23.5% of the investigated animals. Of these, only 20.5% had a perforated ulcer [21]. In an evaluation of SACs examined at the Institute of Veterinary Pathology at the University of Leipzig, Leipzig, Germany, Theuß et al. found inflammatory changes of the stomach in 34% of the animals, but of these, only 2.6% had a perforated ulcer [31]. The alpaca examined in the present case had a perforated ulcer, which resulted in peritonitis and severe blood loss.

According to Neubert et al., gastric ulcers in SACs can be classified into the following four types: 0 = no erosion or ulceration; 1 = erosion(s) (as a preliminary stage of ulceration); 2 = ulceration(s); 3 = perforated gastric ulcer [21]. Thus, in the present case, the lesions in C1 were assessed with a score of 3 and in C3 with a score of 2. However, the red blood count as well as the occult blood test do not appear to be suitable for diagnosing or excluding a gastric ulcer in SACs [20, 21, 37]. In the animal described here, intraluminal blood loss was reflected in pale mucous membranes and a highly decreased haematocrit. Although no occult blood test was performed, the large amounts of digested blood in the digestive tract suggest that this would have led to a positive result.

Evidence of other causes of anaemia could not be found, as both in the faecal sample taken shortly before the animal died and the pathological examination revealed no evidence for *H. contortus*. No clear statement can be made about the character of the anaemia on the basis of the findings from the laboratory of the clinic, as no erythrocyte count was determined and MCH and MCV could therefore not be calculated. It also remains questionable whether the anaemia was regenerative or non-regenerative, as no reticulocytes were determined. Reference values for nucleated red blood cells, which can be released precipitously in highly regenerative anaemia [38], are given as 0–3% for alpacas [5], so that a strong increase in nucleated red blood cells was not detected here. The role of Cabot rings, which are regularly found in the erythrocytes of SACs, is not clear yet [1], but they can be observed in higher numbers in regenerative anaemia [8]. The neutrophil-to-lymphocyte ratio (NLR) in this animal was above the reference value given by Hajduk for alpacas [4]. While not much is known about the NLR in alpacas, there is evidence in other species that it increases with stress [39–41]. The increase in NLR in this alpaca could therefore also be interpreted as an indication of stress. However, since the perforated ulcer itself may have been a major stressor for the animal and the NLR can also change within a short time interval [42], it is questionable whether it can be concluded that stress was the trigger for this clinical picture.

Another description of an alpaca in which large intestinal blood losses occurred was published by Anderson et al. [43]. They found approx. 500 ml of coagulated blood in the lumen of the first compartment of a 14-year-old male alpaca that died peracutely. The cause was an ulcer of the first compartment caused by a squamous cell carcinoma, the third compartment did not reveal any mucosal changes [43]. However, the data provided by Anderson et al. include only pathological data, clinical and laboratory data of the animal are not described, therefore the anaemic condition in this case remains unclear. Tharwat et al. reported the case of a dromedary with the highest degree of anaemia (0.0075 l/l) due to an omaso-abomasal adenocarcinoma [44]. There are also several reports of blood loss due to gastrointestinal neoplasia in other species [45, 46]. Damage to the compartments with subsequent peritonitis was also described by Wallace et al. in connection with gastroliths in a llama [47]. However, neoplasia could not be excluded in the animal described in the present case there, as no histological examination was performed.

Alongside the red blood count, there were also changes in the white blood count, which consisted of leucopaenia with band neutrophils. According to Smith, this is a typical change in SACs with gastric ulcers [20]. In another case of an alpaca with a ruptured gastric ulcer, which the present authors described previously, similar conditions were found [48]. As this animal had been hospitalised for a longer period of time, we observed an ongoing decrease in the number of leucocytes in the peripheral blood. An examination of the abdominal cavity punctate suggested that these had migrated into the abdominal cavity [48]. In contrast to the presented case, the previously described animal did not show anaemia but instead haemoconcentration [48].

Therapy and prophylaxis of gastric ulcers in SAC have already been described in detail elsewhere [32, 48]. A central factor in prophylaxis is the avoidance of stress. Furthermore, proton pump inhibitors like omeprazole can elevate the pH of the third compartment [49]. According to Hund and Wittek, a blood transfusion is also considered a therapeutic option for anaemia [32], which was previously carried out by the local veterinary practice in the case of the alpaca presented here. However, the therapeutic effect is difficult to assess because the PCVs measured before blood transfusion and the next day in the clinic showed no difference (0.13 l/l). It could be assumed that the blood transfusion first led to an increase in PCV, which could not be detected later due to blood loss via the gastric ulcer.

## Conclusions

Contrary to previous statements in the literature, gastric ulcers in SACs can lead to severe anaemia. Clinical signs are pale mucous membranes and a painful abdomen. Bleeding into the gastrointestinal tract can be detected in faeces using rapid occult blood tests, but as not every gastric ulcer is accompanied by bleeding, the test is of limited diagnostic value in SACs [21]. The clinical diagnosis of a gastric ulcer is usually difficult due to mostly nonspecific symptoms [21, 32]. Prophylactically, stress and excessive use of NSAIDs should be avoided.

## Data Availability

All data generated or analysed during this study are included in this published article.

## Abbreviations

AP: Alkaline phophatase
ASAT: Aspratate aminotransferase
CK: Creatine kinase
GLDH: Glutamate dehydrogenase
MCHC: Mean corpuscular haemoglobin concentration
NLR: Neutrophil-to-lymphocyte ratio
NSAID: Non-steroidal anti-inflammatory drug
PCV: Packed cell volume
PMN: Polymorphnuclear neutrophil
SAC: South American camelid
WBC: White blood count
